# Evolution of Vaginal *Candida* spp. over Eight Years: Species Stability and Variability in Antifungal Susceptibility Classifications Using the Integral System YEASTS Plus

**DOI:** 10.3390/jof12100756

**Published:** 2026-10-09

**Authors:** Diana Constanța Pelea, Andreea-Adriana Neamțu, Laura Maghiar, Teodor-Andrei Maghiar, Csaba Nagy, Mădălin Florin Ganea, Olimpia-Daniela Frenț, Corina Beiușanu, Anca Carmen Huniadi, Alin Bodog, Mariana Ganea

**Affiliations:** 1 Preclinic Department, Faculty of Medicine and Pharmacy, University of Oradea, Universității Str., No. 1, 410087 Oradea, Romania; diana_pelea@uoradea.ro (D.C.P.); beiucorina@yahoo.com (C.B.); 2 Department of Toxicology, “Victor Babeș” University of Medicine and Pharmacy, Eftimie Murgu Square, No. 2, 300041 Timișoara, Romania; andreea.neamtu@umft.ro; 3 Research Centre for Pharmaco-Toxicological Evaluation, “Victor Babeș” University of Medicine and Pharmacy, Eftimie Murgu Square, No. 2, 300041 Timișoara, Romania; 4 Department of Pathology, Clinical County Emergency Hospital of Arad, Andrenyi Karoly Str., No. 2–4, 310037 Arad, Romania; 5 Department of Pathology, “Pius Brînzeu” Clinical County Emergency Hospital Timișoara, Liviu Rebreanu Boulevard, No. 156, 300723 Timișoara, Romania; 6 Department of Psycho-Neurosciences and Rehabilitation, Faculty of Medicine and Pharmacy, University of Oradea, Universității Str., No. 1, 410087 Oradea, Romania; 7 Department of Dermatovenerology, Clinical County Emergency Hospital Bihor, 410169 Oradea, Romania; 8 Department of Surgical Sciences, Obstetrics and Gynecology, Faculty of Medicine and Pharmacy, University of Oradea, Universității Str., No. 1, 410087 Oradea, Romania; ahuniadi@uoradea.ro (A.C.H.); alin.bodog@didactic.uoradea.ro (A.B.); 9 Department of Surgery, Pelican Hospital, Corneliu Coposu Str., No. 2, 410450 Oradea, Romania; 10 Doctoral School of Biomedical Sciences, University of Oradea, Universității Str., No. 1, 410087 Oradea, Romania; nagy.csaba@student.uoradea.ro; 11 Department of Pharmacy, Faculty of Medicine and Pharmacy, University of Oradea, Nicolae Jiga Str., No. 29, 410028 Oradea, Romania; ganea.madalinflorin@student.uoradea.ro (M.F.G.); ofrent@uoradea.ro (O.-D.F.); mganea@uoradea.ro (M.G.); 12 Department of Obstetrics and Gynecology, Calla—Infertility Diagnostic and Treatment Center, Constantin A. Rosetti Street, 410103 Oradea, Romania; 13 Department of Embryology, Calla—Infertility Diagnostic and Treatment Center, Constantin A. Rosetti Street, 410103 Oradea, Romania

**Keywords:** vulvovaginal candidiasis, *Candida albicans*, non-albicans *Candida*, antifungal susceptibility, Integral System YEASTS Plus, laboratory surveillance

## Abstract

Vulvovaginal candidiasis is caused predominantly by *Candida albicans*, but non-albicans species differ in antifungal susceptibility, and surveillance based on commercial susceptibility systems must be read against their analytical limitations. We retrospectively analyzed 2789 vaginal *Candida* isolates from 2789 unique patients recovered at Pelican Hospital, Oradea, Romania, between August 2018 and July 2026, tested with the Integral System YEASTS Plus colorimetric system and grouped into four two-year periods. Classifications for clotrimazole, econazole, miconazole, fluconazole and nystatin were analyzed as recorded, without conversion into EUCAST categories. *C. albicans* accounted for 84.6% of isolates, *Nakaseomyces glabratus* (*Candida glabrata*) for 8.4%, *Pichia kudriavzevii* (*Candida krusei*) for 3.8% and the *C. parapsilosis* complex for 2.8%, and the distribution of species assignments did not change across periods (*p* = 0.532). Phenotypes were strongly species dependent: *P. kudriavzevii*, which is intrinsically resistant to fluconazole, was never classified as fluconazole-susceptible (0/107); only 37.2% of *C. parapsilosis* complex isolates were classified as nystatin-susceptible; and 97.3% of *C. albicans* isolates were classified as fluconazole-susceptible, without temporal change. Directional trends surviving multiplicity correction were confined to *C. albicans* but reflected a synchronous final-period rise across pharmacologically distinct agents, while clotrimazole classifications fell and recovered simultaneously in all three non-albicans species during 2020–2022, unlike econazole. These structures, and the progressive enrichment of later periods with newly included patients inherent to the first-isolate design, are more consistent with analytical variation and cohort structure than with resistance evolution. Commercial colorimetric classifications should not be equated with reference MIC-based categories, and the species–drug signals identified require confirmation by reference methods.

## 1. Introduction

Vulvovaginal candidiasis (VVC) is among the most common fungal mucosal disorders affecting women and remains predominantly associated with *Candida albicans*. Non-albicans *Candida* species nevertheless contribute substantially to the clinical complexity of VVC, because species-specific biological characteristics may influence antifungal susceptibility, persistence, recurrence, biofilm formation and therapeutic response [1,2,3,4,5,6,7,8]. The condition carries a substantial global burden and is addressed by current international and national management guidelines, which recommend topical imidazoles, nystatin and oral fluconazole as first-line options [9,10,11,12].

The epidemiology of VVC is heterogeneous across geographical regions and patient populations. Although *C. albicans* generally remains predominant, variable contributions of *Nakaseomyces glabratus* (formerly *Candida glabrata*), *Pichia kudriavzevii* (formerly *Candida krusei*), the *C. parapsilosis* species complex and other species have been reported [1,2,3,4,5,6,7,8]. Local longitudinal surveillance is therefore valuable, because species distributions and phenotypic patterns observed in one population cannot necessarily be extrapolated to another. For Romania, published data on vaginal *Candida* are limited to a recent single-year obstetric and gynecological series [13], and no multi-year surveillance of species distribution or antifungal phenotypes has been reported. Long-term single-centre surveillance has previously been used to characterize the epidemiology of other infections in the same county over a comparable eight-year window [14], and retrospective analyses of clinical and laboratory records from the study hospital have likewise been reported [15]. The same approach is applied here to vaginal yeast isolates, as part of a wider programme of clinical and laboratory research on women’s health conducted by this group [16,17,18].

Antifungal susceptibility testing in VVC presents additional methodological challenges. Standardized reference methods provide reproducible quantitative measurements, whereas antifungal activity at the vaginal mucosal surface may additionally be influenced by local drug concentrations, formulation characteristics, mucosal retention, vaginal pH, fungal biofilm formation, epithelial interactions and the composition of the protective vaginal microbiota.

Commercial colorimetric systems offer a practical approach to yeast identification and phenotypic susceptibility testing in routine clinical microbiology laboratories. Integral System YEASTS Plus has been evaluated against conventional identification and susceptibility methodologies on two occasions, and neither evaluation was favourable: categorical agreement between the predecessor panel and reference broth microdilution was 37.6% [19], and the current version misidentified or failed to code more than half of the isolates tested, with identification agreement of only 5% for non-albicans species and miconazole the least reliable agent on the panel [20]. The system has more recently been used together with broth microdilution and biofilm assessment for genital yeast isolates [1]. Categorical classifications generated by commercial systems and quantitative results obtained by standardized broth microdilution are not analytically interchangeable. Reference susceptibility testing relies on standardized MIC determination and, where available, species-specific and antifungal-specific clinical breakpoints or epidemiological cut-off values [21,22,23]. Retrospective commercial-system classifications should therefore not be converted into EUCAST susceptible, intermediate or resistant categories without appropriate methodological validation.

Despite these limitations, longitudinal analysis of results generated consistently by the same commercial methodology can provide clinically relevant epidemiological information. Such surveillance can identify shifts in species distribution, temporal variation in phenotypic profiles and species–antifungal combinations that warrant prospective investigation. It can also, when analyzed critically, reveal patterns that are informative about the analytical stability of the laboratory process itself. This second use is frequently neglected in retrospective susceptibility surveillance, yet it is essential: a temporal change in reported susceptibility is only interpretable as a change in the organisms if the measurement process can be shown to have remained stable.

The aim of this study was to describe the evolution of vaginal *Candida* species assignments and antifungal susceptibility classifications generated by a single commercial system over eight consecutive years at Pelican Hospital, Oradea, Romania, using 2789 isolates from 2789 unique patients. Specifically, we asked (i) whether the distribution of species changed between 2018 and 2026; (ii) how susceptibility classifications differed between species and across two-year periods; and (iii) whether the temporal signals observed were consistent with biological change or with analytical variation, in order to identify the species–drug combinations that warrant prospective reference-method testing. The third question was addressed with post hoc internal consistency analyses introduced explicitly as a safeguard against overinterpretation.

## 2. Materials and Methods

### 2.1. Study Design and Setting

A retrospective laboratory-based observational study was conducted at Pelican Hospital, Oradea, Romania. Laboratory records covering the eight-year period from August 2018 through July 2026 were evaluated. The study was conducted within the research protocol “Evaluation of the susceptibility of bacterial and fungal strains to antimicrobials”, approved by the Ethics Committee for Scientific Research of Pelican Hospital (Pelican Impex S.R.L.), Oradea, Romania, decision number I02230 of 12 December 2017, authorizing the study from 1 January 2018 to 31 December 2030 in accordance with applicable data protection regulations. The requirement for individual informed consent was waived by the Ethics Committee because the study used only pre-existing, anonymized laboratory records generated during routine diagnostic activity. All data were processed without disclosure of patient identity.

### 2.2. Study Population and Isolate Selection

Vaginal *Candida* isolates recovered during routine microbiological investigation were eligible for inclusion. Eligibility was defined microbiologically: symptom status, clinical diagnostic criteria and the indication for sampling were not recorded in the dataset, so the cohort represents women from whom *Candida* was recovered rather than women with a clinical diagnosis of vulvovaginal candidiasis. A unique-patient principle was applied throughout: the first chronologically eligible *Candida* isolate from each unique patient was retained, and no patient contributed more than one isolate over the entire surveillance period. Patients were matched across the eight years using the unique patient identifier held in the laboratory information system. This prevented repeated sampling of the same patient from disproportionately influencing species frequencies or susceptibility estimates. The consequences of this design choice for the interpretation of temporal trends are addressed in Section 4.6 and Section 5.

During the surveillance period the laboratory performed 17,267 vaginal fungal cultures. Fungal culture was occasionally requested alone, but in most cases formed part of a panel alongside bacterial culture, culture for *Ureaplasma* spp. and *Mycoplasma hominis*, and microscopic examination. Genital mycoplasmas were detected with a commercial dehydrated liquid-medium culture system (Mycoplasma IES, ref. M0205, Autobio Diagnostics Co., Ltd., Zhengzhou, China; CE-IVD), in which growth of *Ureaplasma* spp. or *M. hominis* is detected by a pH-indicator colour change in the broth, not by cell culture. Sampling was therefore frequently not driven by a clinical suspicion of candidiasis specifically, which is relevant to the interpretation of the cohort (Section 5). No specimen in the compiled dataset was recorded as yielding more than one yeast species, so no rule for handling mixed cultures was required. The final analytical cohort comprised 2789 isolates from 2789 unique patients, corresponding to 16.2% of all vaginal fungal cultures performed; because repeated positive specimens from the same woman were collapsed to a single isolate, this proportion is lower than the true specimen-level positivity rate, which cannot be recovered from the aggregated dataset. For longitudinal analysis the observation period was divided into four consecutive intervals: August 2018 to July 2020, August 2020 to July 2022, August 2022 to July 2024 and August 2024 to July 2026, containing 512, 562, 749 and 966 isolates, respectively.

### 2.3. Laboratory Procedure

Yeast identification and antifungal susceptibility testing were performed with the Integral System YEASTS Plus commercial colorimetric system (Liofilchem, Roseto degli Abruzzi, Italy; ref. 71822) throughout the surveillance period, strictly according to the manufacturer’s instructions and without modification of the recommended procedure. The panel comprises 24 reaction wells, of which wells 14 to 23 contain antimycotics, and is incubated at 36 degrees Celsius for 48 h [24]. Each antifungal is present in a single well at one fixed concentration. According to the package insert (revision 4, dated 25 July 2012), the concentrations relevant to this study are nystatin 1.25, clotrimazole 1, econazole 2, miconazole 2 and fluconazole 64 µg/mL [25]; the remaining wells contain amphotericin B, flucytosine, ketoconazole, itraconazole and voriconazole, which were not part of the reported dataset. The antifungal wells are red before inoculation. After incubation, a well that remains red indicates inhibition of growth and therefore susceptibility to the concentration present in the well, a shift to orange indicates intermediate susceptibility, and a shift to yellow indicates resistance [24,25]. Revision 4 of the package insert is the version held in the laboratory’s kit documentation, and the lot in use at the end of the surveillance period (lot 032125513, expiry 9 July 2026) was still supplied with revision 4, so no later revision was issued with the kits used during the study; each lot is also accompanied by a manufacturer’s certificate of analysis documenting identification and susceptibility performance with a reference *C. albicans* strain. This nominal continuity of the testing protocol is what permits internal longitudinal comparison of commercial-system phenotypes; it does not by itself establish analytical stability, as the final paragraph of this section and Section 4.5 make clear.

The principal species recorded in the historical laboratory database were *Candida albicans*, *Candida glabrata*, *Candida krusei* and *Candida parapsilosis*, the names generated by the commercial identification system. Less frequently identified species were grouped as other *Candida* spp. for epidemiological analysis. In this manuscript the species are reported under their currently accepted names, *Nakaseomyces glabratus* (formerly *C. glabrata*) and *Pichia kudriavzevii* (formerly *C. krusei*) [26]; the legacy names are given in parentheses at first mention and in table headers because they are the labels generated by the system and held in the historical database. Isolates reported by the system as *C. parapsilosis* are referred to as the *C. parapsilosis* complex, since the system cannot distinguish *C. parapsilosis* sensu stricto from *C. orthopsilosis* and *C. metapsilosis*. Throughout, species names denote commercial-system assignments rather than reference-method identifications.

The commercial colorimetric system does not reliably discriminate *C. albicans* from *C. dubliniensis*. Isolates reported as *C. albicans* in this dataset should therefore be understood as the *C. albicans* and *C. dubliniensis* complex [27].

Two features of laboratory practice over the surveillance period are relevant to the interpretation of temporal results and are stated here rather than as a retrospective caveat. Kit lots were replaced several times a year in the normal course of consumable supply (three lots in 2024 and eight in 2025 in the years for which lot records were retrieved; Supplementary Table S5), and the laboratory personnel performing and reading the tests changed during the 2022 to 2024 interval. Every kit lot is tested on receipt with the reference strain *C. parapsilosis* ATCC 22019 before being placed in use, and the laboratory’s non-conformity register records no quality-control deviation for any lot over the surveillance period; the lot-level acceptance records for 2024 and 2025 were retrieved for this analysis, and those for earlier years are held in the institutional archive. Control results are recorded categorically for a single wild-type strain, so they document that each lot was functional but do not measure the position of each well’s effective endpoint to a fraction of a dilution (Section 4.5), and reading logs identifying the operator of individual results were not retained. Isolates were discarded immediately after testing in accordance with national regulations on the management of waste from medical activities [28], so no strain collection is available for retesting. Routine susceptibility testing of topical antifungal agents with this system was scheduled for discontinuation at the study laboratory from September 2026, so the eight-year series constitutes a closed historical record.

### 2.4. Antifungal Susceptibility Classification

The antifungal agents included in the retrospective dataset were clotrimazole, econazole, miconazole, fluconazole and nystatin. For each species and interval, the number and proportion of isolates classified as susceptible by Integral System YEASTS Plus were calculated.

Throughout this manuscript, isolates were categorized as susceptible versus non-susceptible, the latter comprising all isolates not reported as susceptible by the system. Historical classifications were analyzed exactly as recorded and were not transformed into EUCAST or CLSI categories, because EUCAST requires that a commercial test be qualified against the reference microdilution method before its breakpoints are adopted [29], and no such qualification was performed. Fluconazole is the only agent among the five analyzed for which EUCAST breakpoints are defined; clotrimazole, econazole, miconazole and nystatin appear in neither the EUCAST clinical breakpoint tables nor the EUCAST overview of epidemiological cut-off values for yeasts [22,30]. Accordingly, unless otherwise stated, the terms susceptible and susceptibility refer specifically to classification by Integral System YEASTS Plus and carry no reference-method or clinical resistance meaning.

Integral System YEASTS Plus presents three categories, read colorimetrically as susceptible, intermediate and resistant. In compiling the surveillance dataset, only isolates classified as susceptible were counted as such; intermediate results were not included in the susceptible category and therefore fall within the non-susceptible group together with resistant results. Counts of intermediate and resistant classifications were not recorded separately in the aggregated dataset and cannot be reported individually here. All isolates identified during the period are represented in the denominators, and none were excluded on the basis of an intermediate result.

### 2.5. Statistical Analysis

Categorical variables were summarized as absolute numbers and percentages, with 95% confidence intervals calculated by the Wilson score method, which is appropriate for the small denominators encountered among non-albicans species.

Overall species distribution across the four surveillance periods was evaluated by Pearson chi-square. Because four of the twenty cells in the 5 × 4 table had expected counts below five (minimum 1.84), owing to the rarity of the other *Candida* spp. category, two confirmatory analyses were performed: a Monte Carlo approximation to a conditional exact test with fixed margins based on 50,000 replicates, and a restricted analysis of the four principal species only. Temporal changes in the proportional representation of individual species were evaluated using binomial generalized linear models with a logit link, with period entered as an ordinal score (1 to 4) modelled as a single linear term. The Benjamini–Hochberg procedure was applied separately across the five species-specific proportional trend tests.

For each species–antifungal combination, between-period differences in the proportion classified as susceptible were evaluated using a Monte Carlo approximation to a conditional exact test for 2 × 4 tables with fixed margins, based on 200,000 replicates. This approach was adopted in preference to the asymptotic chi-square test because several non-albicans strata contained fewer than ten isolates per period, and expected counts fell as low as 1.6. The number of replicates was chosen so that the Monte Carlo standard error of a *p* value close to the 0.05 threshold is below 0.0005 (approximately √(0.05 × 0.95/200,000) = 0.00049), which stabilizes the third decimal place of the reported *p* values; re-running the analysis with 50,000, 100,000 and 1,000,000 replicates changed no *p* value by more than 0.005 and no conclusion at the nominal or the adjusted level (Supplementary Table S4). Linear temporal trends were evaluated using binomial generalized linear models based on susceptible and non-susceptible counts in each period, with the trend *p* value taken from the Wald test of the period coefficient. Where all isolates displayed an identical categorical phenotype across every interval, as occurred for *P. kudriavzevii* and fluconazole, inferential testing was not applicable.

Nineteen species–antifungal comparisons were testable. The Benjamini–Hochberg procedure was applied to control the false discovery rate at 5%, that is, the expected proportion of false-positive findings among the comparisons declared significant, separately for the family of between-period tests and the family of trend tests. Adjusted *q* values are reported alongside nominal *p* values. Findings that reached nominal significance but did not retain significance after adjustment are described as exploratory. A two-sided nominal *p* value below 0.05 was considered significant before correction.

Trend models are reported as odds ratios per one-period increment with 95% confidence intervals in Supplementary Table S2, alongside nominal and adjusted *p* values; the complete susceptible and non-susceptible counts underlying every analysis are given in Supplementary Table S1.

Analyses were performed in Python 3.12.3 using NumPy 2.4.4, SciPy 1.17.1 and statsmodels 0.14.6, with figures generated with Matplotlib 3.10.8; the exact versions of all dependencies and the fixed random seed (20260822) are recorded in the header of the analysis code provided as Supplementary Material, which reproduces every test, adjusted *p* value and figure from the counts in Supplementary Table S1. Monte Carlo resampling used the NumPy default random generator.

### 2.6. Post Hoc Internal Consistency Analyses

Two consistency analyses were used to test whether observed temporal signals behaved as biological resistance change would be expected to behave. These analyses were not prespecified: they were formulated after inspection of the period-specific results and are therefore reported as post hoc and exploratory throughout. They are presented because their structure is informative, not as confirmatory hypothesis tests.

First, drug-specificity and species-specificity of any significant temporal signal were examined. A genuine population-level change in susceptibility of one species should not, in general, appear simultaneously and abruptly across pharmacologically distinct agents within a single interval, whereas a change in the measurement process may do so. Conversely, a change affecting the assay itself would be expected to affect multiple species tested on the same panel.

Second, the *P. kudriavzevii* fluconazole result was examined descriptively as an invariant commercial-system phenotype. The analysis assessed whether susceptible calls emerged over time; it was not treated as a biological or analytical control.

## 3. Results

### 3.1. Overall Distribution of Vaginal Candida Species

A total of 2789 vaginal *Candida* isolates from 2789 unique patients were included. *C. albicans* was predominant, accounting for 2359 isolates (84.6%, 95% CI 83.2 to 85.9). Non-albicans *Candida* collectively accounted for 430 isolates (15.4%, 95% CI 14.1 to 16.8): *N. glabratus* (*C. glabrata*) 235 (8.4%, 95% CI 7.5 to 9.5), *P. kudriavzevii* (*C. krusei*) 107 (3.8%, 95% CI 3.2 to 4.6), *C. parapsilosis* complex 78 (2.8%, 95% CI 2.2 to 3.5) and other *Candida* spp. 10 (0.4%, 95% CI 0.2 to 0.7). Confidence intervals were calculated by the Wilson score method.

### 3.2. Temporal Stability of Species Distribution

**Figure 1:**
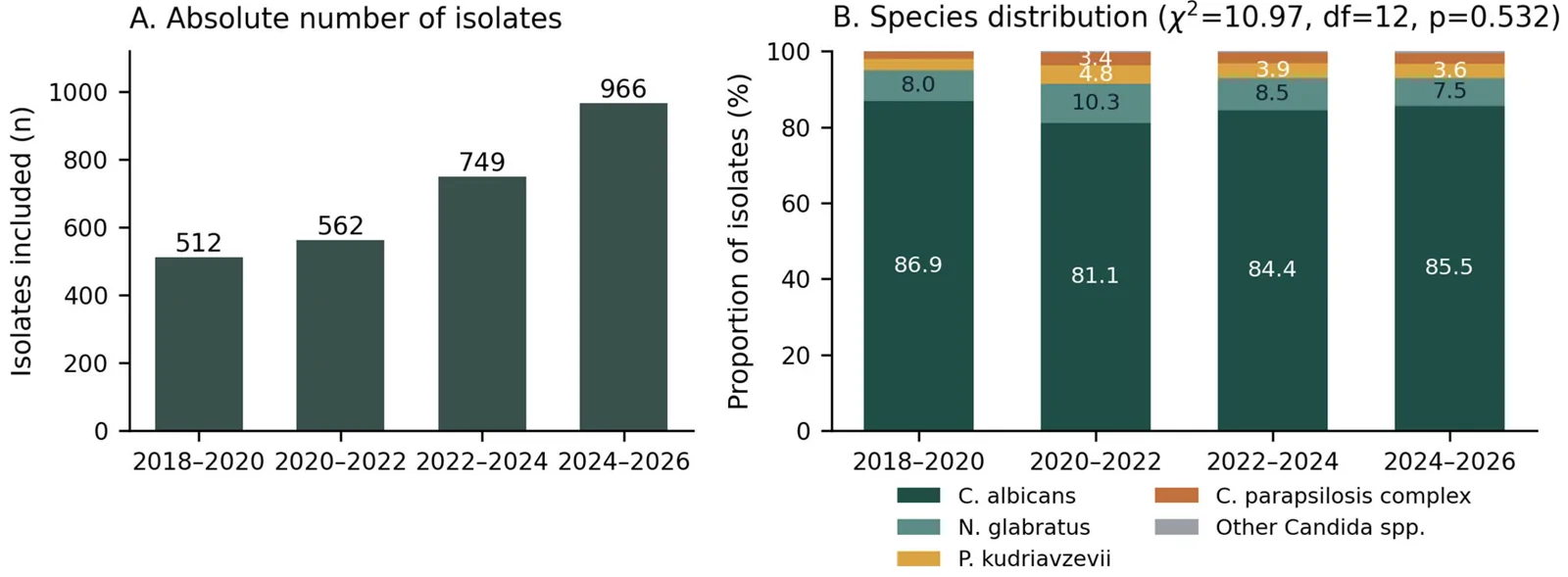
**Distribution of vaginal Candida species during the eight-year surveillance period.** (**A**) Absolute number of isolates included in each two-year interval under the unique-patient principle. (**B**) Proportional distribution of commercial-system species assignments, which remained statistically stable across the four intervals. The contrast between panels (**A**) and (**B**) is central to interpretation: absolute counts nearly doubled while relative composition did not change.

The absolute number of included isolates increased across the four intervals from 512 to 562, 749 and 966 (Figure 1A). Although 17,267 vaginal fungal cultures were performed over the whole period, the number of cultures performed within each individual interval and the underlying population denominators were unavailable, so this increase must not be interpreted as evidence of rising VVC incidence or prevalence; it more plausibly reflects laboratory workload and referral patterns.

Species proportions are shown in Table 1 and Figure 1B. Overall distribution did not differ significantly across the four periods (χ^2^ = 10.97, df = 12, asymptotic *p* = 0.532). Because four cells had expected counts below five, this result was confirmed by a Monte Carlo approximation to a conditional exact test, which returned an identical estimate (*p* = 0.532), and by a restricted analysis limited to the four principal species, in which all expected counts exceeded 14 (χ^2^ = 9.72, df = 9, *p* = 0.373). The conclusion is therefore not an artefact of sparse cells.

No species showed a significant proportional trend across periods, either nominally or after false discovery rate adjustment (all *q* ≥ 0.648). Despite the substantial increase in absolute isolate numbers, the relative distribution of commercial-system species assignments remained statistically stable throughout the eight-year period.

### 3.3. Aggregate Species-Specific Antifungal Phenotypes

Aggregate proportions classified as susceptible are given in Table 2. Among the 2359 *C. albicans* isolates, fluconazole showed the highest aggregate proportion classified as susceptible (97.3%, 95% CI 96.6 to 97.9). Among 235 *N. glabratus* isolates, values clustered between 75.7% and 82.1% across all five agents. Among 107 *P. kudriavzevii* isolates, no isolate was classified as fluconazole-susceptible at any point (0/107, 95% CI 0.0 to 3.5), as expected from the intrinsic fluconazole resistance of this species, while miconazole showed the lowest classification among the remaining agents (58.9%). Among 78 *C. parapsilosis* complex isolates, the proportion classified as nystatin-susceptible was 37.2% (95% CI 27.3 to 48.3), the most distinctive aggregate phenotype in the dataset. These two species-restricted phenotypes are highlighted in Table 2.

### 3.4. Temporal Susceptibility Classifications

Period-specific counts, conditional between-period tests, trend tests and false discovery rate adjusted *q* values for all 20 species–antifungal combinations are presented in Table 3.

### 3.5. Temporal Profile of C. albicans

Among *C. albicans*, fluconazole classifications remained consistently high throughout: 97.3%, 96.1%, 96.8% and 98.3%. Neither the between-period comparison (Monte Carlo conditional *p* = 0.094) nor the linear trend (*p* = 0.117, *q* = 0.278) approached significance. The eight-year dataset therefore provides no evidence of deterioration in the reported fluconazole phenotype of vaginal *C. albicans*. This is a statement about the stability of the classification over time and not about the prevalence of clinically relevant resistance: fluconazole is tested at a single concentration of 64 µg/mL, far above the EUCAST breakpoints for this species, so the panel cannot detect resistance below that concentration and the high proportion partly reflects a ceiling effect (Section 4.3).

**Figure 2:**
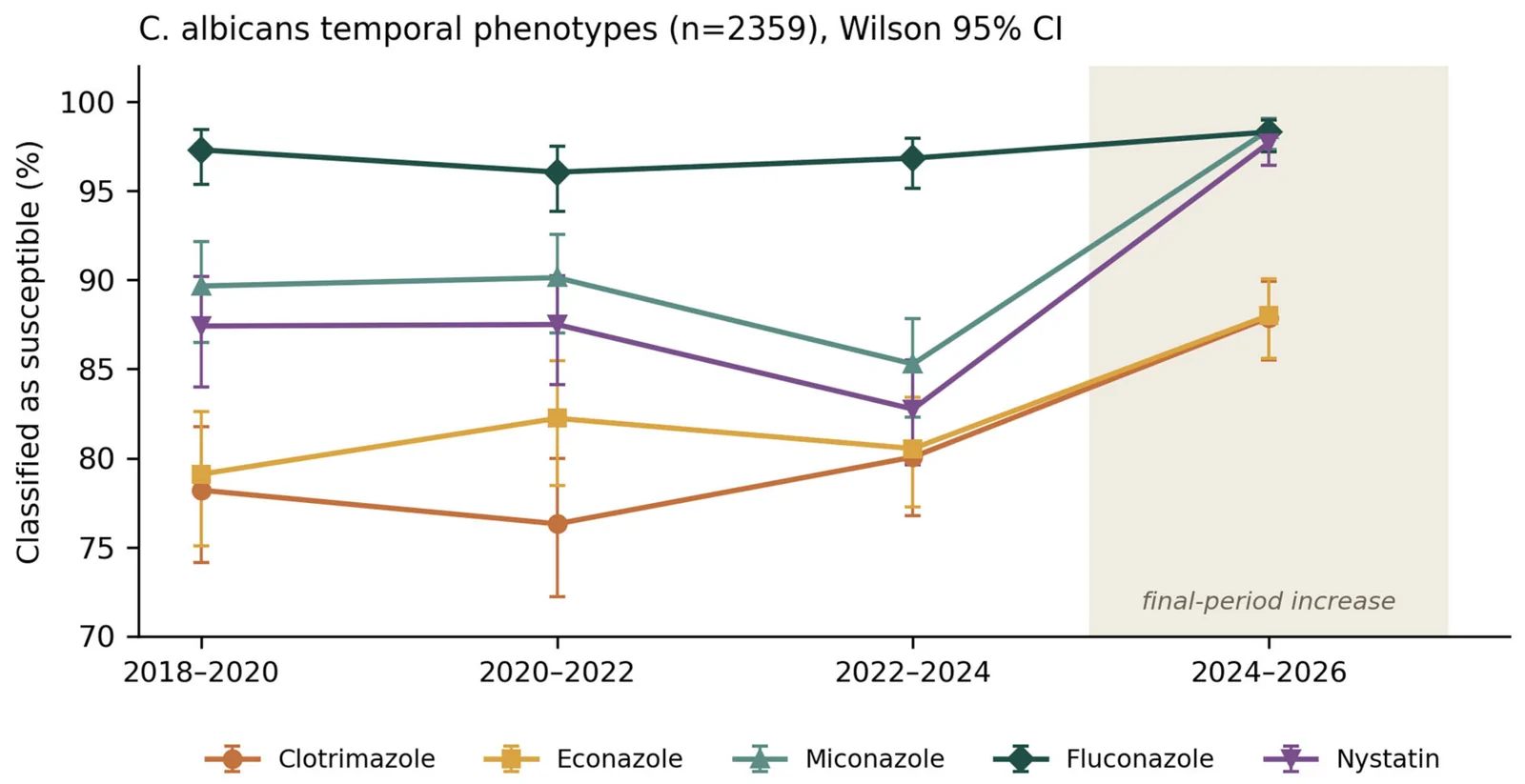
**Temporal antifungal susceptibility profile of** ***Candida albicans*****.** Proportions classified as susceptible across the four surveillance periods, with Wilson 95% confidence intervals. Fluconazole remained stable. The four remaining agents converge sharply in the final interval, shaded. The simultaneity of this convergence across pharmacologically distinct agents is the observation examined in Section 3.7.

Significant between-period variation was observed for clotrimazole, econazole, miconazole and nystatin, and all four retained significance after false discovery rate adjustment (all *q* < 0.001). Clotrimazole rose from 78.2% to 87.9% and econazole from 79.1% to 88.0%. Miconazole classifications were 89.7%, 90.1%, 85.3% and 98.4% in the four successive periods, and nystatin classifications were 87.4%, 87.5%, 82.8% and 97.7% (Figure 2). The latter two trajectories are non-monotonic, with a decline during 2022 to 2024 followed by a pronounced rise in the final interval. Rather than a progressive improvement, these patterns represent significant heterogeneity between periods that is dominated by an increase in the final interval. Their interpretation is qualified by the consistency analysis in Section 3.7.

### 3.6. Temporal Profiles of Non-Albicans Candida

Among *N. glabratus*, between-period differences were significant for clotrimazole (Monte Carlo conditional *p* < 0.001, *q* < 0.001), driven by the second-interval decline described above. The nominal trends for clotrimazole (*p* = 0.016) and miconazole (*p* = 0.042) did not survive false discovery rate adjustment (*q* = 0.059 and *q* = 0.133) and are therefore exploratory. Econazole, fluconazole and nystatin showed neither between-period differences nor trends.

Among *P. kudriavzevii*, clotrimazole differed significantly between periods and retained significance after adjustment (*q* = 0.004), again reflecting the second-interval decline, while no linear trend was demonstrated (*q* = 0.174). Econazole reached nominal between-period significance (*p* = 0.045) but not adjusted significance (*q* = 0.106). Fluconazole remained uniformly non-susceptible.

Among *C. parapsilosis*, clotrimazole differed between periods nominally (*p* = 0.023) but not after adjustment (*q* = 0.062). No agent showed a statistically robust temporal trend. The nystatin sequence, 44.4%, 36.8%, 18.2% and 50.0%, was highly variable but showed neither a significant between-period difference (*q* = 0.257) nor a trend (*q* = 0.662), and rests on denominators of 9 to 28 isolates.

**Figure 3:**
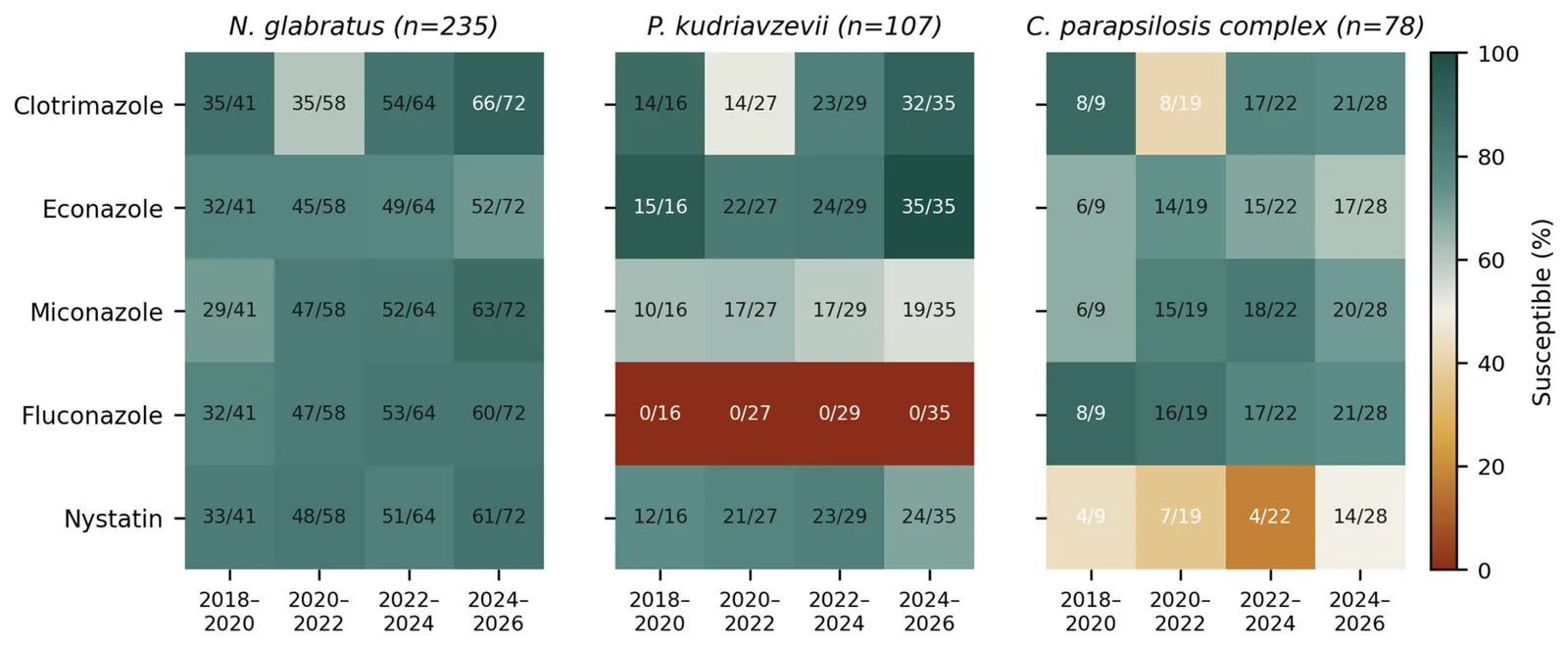
**Temporal antifungal susceptibility patterns among non-albicans Candida.** Heatmaps showing susceptible over total isolates for each species, agent and interval, with colour encoding the corresponding percentage. The *P. kudriavzevii* fluconazole row was uniformly non-susceptible across all intervals. Apparent temporal patterns should be interpreted cautiously in view of the denominators shown, the multiplicity adjustment in Table 3 and the consistency analysis in Section 3.7.

Given the small numbers of non-albicans isolates, particularly *C. parapsilosis*, these findings should be interpreted descriptively (Figure 3). The internal consistency of these temporal patterns, and of those observed in *C. albicans*, is examined in Section 3.7.

### 3.7. Post Hoc Internal Consistency of the Temporal Signals

The two post hoc consistency analyses described in Section 2.6 produced three findings that materially qualify the temporal results.

Final-period convergence in *C. albicans*. All four *C. albicans* agents with statistically robust trends increased simultaneously in 2024 to 2026, and the two largest increases, miconazole (85.3% to 98.4%) and nystatin (82.8% to 97.7%), occurred for agents from different pharmacological classes. Fluconazole did not rise appreciably, but it was already at 96.8% and had almost no headroom. Critically, this convergence was restricted to *C. albicans*: among pooled non-albicans isolates tested on the same panel over the same interval, miconazole classifications were 75.7% and then 75.6%, and nystatin 67.8% and then 73.3%, with no comparable shift.

**Figure 4:**
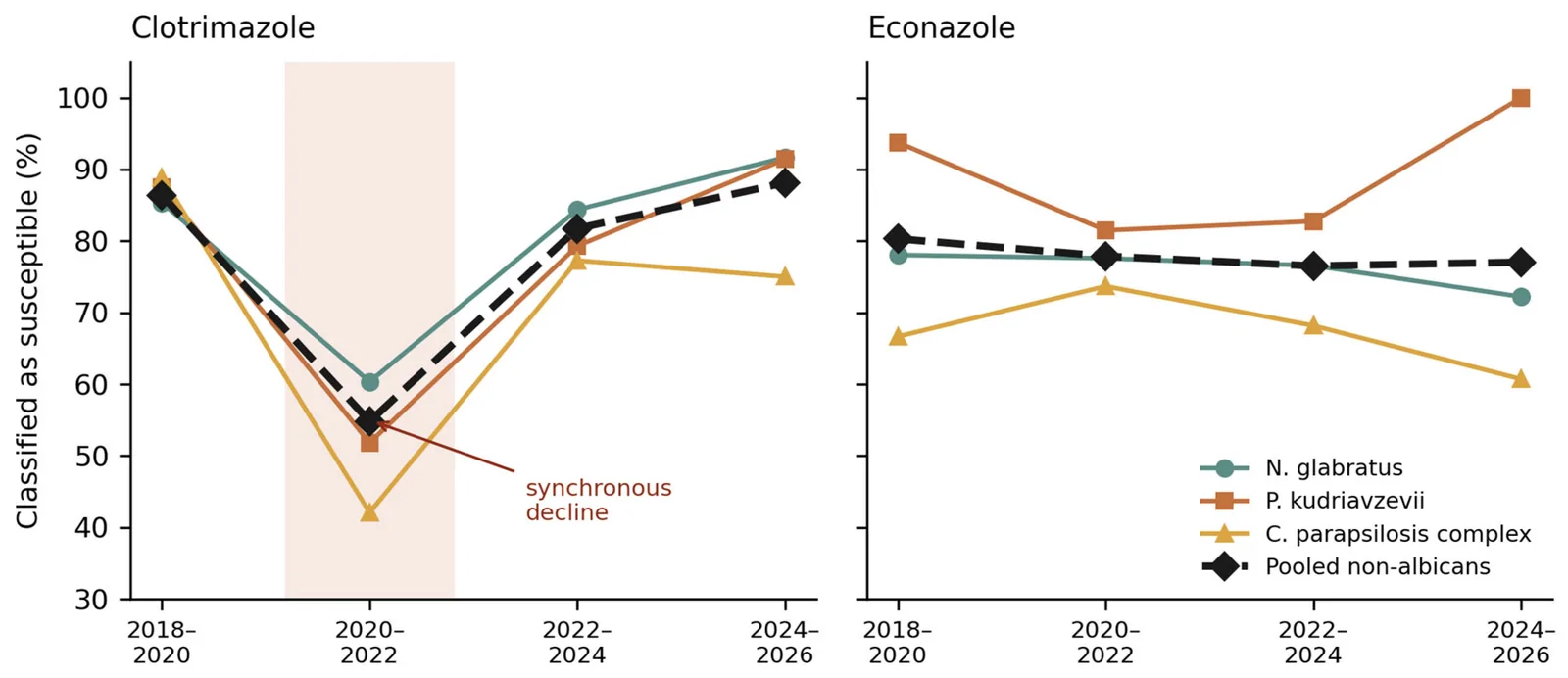
**Synchronous clotrimazole anomaly among non-albicans Candida during 2020 to 2022.** (**Left**), clotrimazole classifications in *N. glabratus*, *P. kudriavzevii* and the *C. parapsilosis* complex, with the pooled non-albicans value, showing a simultaneous decline in the second interval and full recovery thereafter. (**Right**), econazole classifications over the same intervals in the same isolates, showing no corresponding change. The pooled non-albicans value is the proportion classified as susceptible among all isolates of the three non-albicans species combined in each interval (*n* = 66, 104, 115 and 135 isolates in the four successive periods).

Synchronous non-albicans clotrimazole decline in 2020 to 2022. Clotrimazole classifications fell simultaneously in all three non-albicans species during the second interval and then recovered: *N. glabratus* 85.4% to 60.3% to 84.4% to 91.7%, *P. kudriavzevii* 87.5% to 51.9% to 79.3% to 91.4%, and *C. parapsilosis* 88.9% to 42.1% to 77.3% to 75.0%. Pooled across the three species the sequence was 86.4%, 54.8%, 81.7% and 88.1%. No parallel change occurred for econazole, structurally the closest comparator agent on the same panel, which remained between 76.5% and 80.3% throughout (Figure 4). A biological explanation would require three independently circulating species to lose and then regain clotrimazole susceptibility in unison over two years, while remaining unchanged for a closely related imidazole.

Invariant commercial-system phenotype. The *P. kudriavzevii* fluconazole phenotype was invariant at 0/107 across all four intervals, providing no evidence that the classification pipeline drifted in a way that would generate spurious fluconazole-susceptible calls among isolates assigned to this species. It does not independently validate the accuracy of that species assignment, which the identification performance described in Section 4.1 leaves open.

Taken together, these analyses show internal coherence in the classification patterns examined here, but the temporal susceptibility signals display drug-restricted and period-restricted structures that biological resistance dynamics do not readily explain. They are compatible with analytical or process variation and with cohort-structure effects, rather than providing evidence of biological resistance evolution. The implications are developed in Section 4.5.

Alignment with laboratory quality records. Acceptance testing of every kit lot with *C. parapsilosis* ATCC 22019 recorded no deviation over the surveillance period, and lots turned over several times a year in the years for which lot records were retrieved (Section 2.3). Two consequences follow. A gross lot failure, such as a well lacking active drug, would have been detected at acceptance and is excluded as an explanation for any of the temporal signals. Conversely, because lots changed several times within each two-year interval, neither the 2020 to 2022 clotrimazole anomaly nor the 2022 to 2024 miconazole and nystatin trough can be attributed to a single lot: an explanation in terms of reagents would require a sustained shift across a series of lots, and an explanation in terms of reading would require a change affecting the interpretation of the corresponding wells over the whole interval. What acceptance testing with a single wild-type strain, read categorically, cannot detect is a displacement of the effective endpoint by a fraction of a dilution, which is the mechanism examined in Section 4.5.

## 4. Discussion

### 4.1. Analytical Validity of the Commercial System Is the Premise for Everything That Follows

Because every observation reported here derives from a single commercial colorimetric system, the documented performance of that system determines how each finding may be read. We therefore address it first rather than as a closing caveat.

Integral System YEASTS Plus and its predecessor have been evaluated against reference broth microdilution on two occasions, and neither evaluation was favourable. In a multicentre Italian study of 793 clinical *Candida* isolates, categorical agreement between the predecessor system and the NCCLS M27-A reference method was 37.6% overall and 39.1% for *C. albicans*; the system classified 40.6% of *C. albicans* isolates as fluconazole-susceptible against 90.1% by the reference method, intralaboratory reproducibility was 71%, and the authors attributed the discrepancies to subjective colorimetric endpoint reading and to the absence of inoculum and medium standardization [19]. In the subsequent evaluation of the current version, 53.4% of isolates were misidentified relative to conventional methods or received no interpretable code; identification agreement was 91.1% for *C. albicans* but 5% for non-albicans species, with 28 of 34 non-albicans isolates reported as *C. albicans*; and receiver operating characteristic analysis gave areas under the curve of 0.28 to 0.56 across antifungals, with miconazole performing worst [20]. Both studies recommended alternative systems. The manufacturer’s documentation still cites NCCLS M27-A2, published in 2002, as the methodological standard for the susceptibility component of the panel [24,31]; that standard and its interpretive supplements have been superseded repeatedly since [21,32], which is a further reason why these classifications should not be mapped onto contemporary EUCAST or CLSI categories.

These limitations are not confined to this product. Substantial MIC variation has been reported across commercial antifungal tests [33], and EUCAST has formalized what a laboratory must demonstrate before applying EUCAST breakpoints to any commercial test: quality control strains are run repeatedly and the modal MIC must correspond to the target, systematic deviation of the mode is explicitly disqualifying and requires investigation of drug concentrations, plasticware or endpoint reading, and a confirmatory panel of unrelated clinical isolates follows [29]. Two features of this guidance bear on the present study. It names endpoint reading among the candidate explanations for systematic deviation, the same mechanism identified in both published evaluations and invoked in Section 4.5, and it establishes that categorical output from an unqualified commercial test cannot carry EUCAST meaning, which is why the terminology of this manuscript is restricted to classification by Integral System YEASTS Plus.

A further consideration applies to topical agents. Clotrimazole, econazole, miconazole and nystatin are absent from both the EUCAST clinical breakpoint tables and the EUCAST overview of epidemiological cut-off values [22,30], so for four of the five agents examined here there is no reference standard at all. Even where a breakpoint exists, EUCAST clinical breakpoints are constructed for defined systemic dosing regimens [22], not for topically applied agents, whose concentration at the vaginal mucosal surface bears no fixed relationship to systemic exposure. For these agents the term susceptible has no reference-method meaning to be borrowed, a limitation that constrains every study reporting categorical susceptibility of topical antifungals in VVC. Three consequences follow for the present data and are developed in Section 4.2, Section 4.3 and Section 4.4. A fourth, developed in Section 4.5, is that both evaluations identify subjective visual endpoint reading as the dominant source of misclassification, which provides a documented mechanism for the agent-restricted and interval-restricted temporal patterns described in Section 3.7.

### 4.2. Commercial-System Species Assignments Were Stable, but Their Absolute Distribution Is Method Dependent

The principal epidemiological finding is stability. *C. albicans* represented approximately 85% of assignments and non-albicans species approximately 15%, and despite a near doubling of absolute isolate numbers neither the overall distribution nor the relative contribution of any species shifted significantly, under conditional testing and after exclusion of sparse categories.

The absolute level must be read against the identification performance described above. A system that reports most non-albicans isolates as *C. albicans* inflates the apparent dominance of that species, and the direction of the bias is known. The present cohort sits well above contemporary series using MALDI-TOF or molecular identification, which reported 63.7% [8] and 50% *C. albicans* [7]. The only other Romanian vaginal series, a single-year obstetric and gynecological study from Iași using chromogenic media with VITEK 2 confirmation, reported 55.3% *C. albicans* among 2020 vaginal discharge isolates, with *P. kudriavzevii* (26.6%) far exceeding *N. glabratus* (4.1%) [13]. The reverse ranking in the present dataset, *N. glabratus* 8.4% and *P. kudriavzevii* 3.8%, illustrates how strongly the reported non-albicans composition depends on the identification method, and no multi-year Romanian surveillance exists against which either series could be tested. We therefore do not claim that vaginal *Candida* ecology in this population is more strongly *C. albicans* dominated than elsewhere; the species proportions reported here are commercial-system assignments, and the true non-albicans fraction is likely to be higher.

The stability finding is affected differently. A misidentification bias that is constant across the eight years displaces the level of every interval by a similar amount and leaves the between-period comparison largely intact, which is why we advance the stability conclusion while withholding the prevalence conclusion. Two qualifications remain. A system with 5% identification agreement for non-albicans species is poorly placed to detect a shift toward non-albicans species, so the absence of such a shift here does not contradict reports of one elsewhere. Likewise, a change in composition within the non-albicans group, for instance an increase in the *C. parapsilosis* complex at the expense of *N. glabratus*, could be masked if part of each species is misassigned to *C. albicans*; the assigned labels showed no such trend (Table 1), but they are an insensitive instrument for detecting one, and the stability conclusion is therefore confined to the assignments themselves.

### 4.3. The C. albicans Fluconazole Phenotype and the Ceiling Imposed by the Assay

Among *C. albicans*, 97.3% of isolates were classified as fluconazole-susceptible, with interval values between 96.1% and 98.3% and no significant between-period difference or trend. Taken at face value, this indicates no progressive deterioration in the reported fluconazole phenotype between 2018 and 2026.

The value itself is a property of the panel design. Each antifungal is presented in a single well at one fixed concentration rather than as a dilution series, so the panel cannot generate a minimum inhibitory concentration; it reports only whether growth was inhibited at that concentration, and every isolate below it is indistinguishable. For fluconazole the package insert specifies 64 µg/mL [25], sixteen times the EUCAST resistance breakpoint of 4 mg/L, thirty-two times the susceptible breakpoint of 2 mg/L and one hundred and twenty-eight times the wild-type epidemiological cut-off of 0.5 mg/L [22,30]. Isolates with reference-method MICs above the EUCAST resistance breakpoint but below 64 µg/mL are therefore classified as susceptible together with wild-type isolates. This ceiling effect provides a plausible mechanism for the very major errors reported for this system, in which isolates resistant by broth microdilution were classified as susceptible [20], and for the very high proportion observed here; because reference MICs were not available for the present isolates, it cannot be demonstrated directly. The published evaluation of the system reported fluconazole at 16 and 64 mg/L [20], whereas revision 4 of the package insert, the version used throughout the surveillance period, specifies a single well at 64 mg/L [25]; the discrepancy most likely reflects a different revision or variant of the panel.

Two conclusions follow, and they point in opposite directions. The negative finding is informative about the stability of the measurement: a phenotype near the ceiling of its scale has little room to rise and considerable room to fall, so a substantial downward shift in the commercial-system classification would have been detectable across the eight years. It is not informative about the prevalence of clinically relevant fluconazole resistance, which this configuration cannot measure; local epidemiological cut-off values determined by reference methodology are the appropriate instrument for that question [23].

### 4.4. Species-Specific Phenotypes: Informative Contrasts and the Invariant P. kudriavzevii Phenotype

The results demonstrate why pooled genus-level susceptibility percentages mislead. Fluconazole provides the clearest example: 97.3% of *C. albicans* isolates were classified as susceptible, whereas none of 107 *P. kudriavzevii* isolates was so classified in any interval. A comparable observation applies to nystatin, favourable among *C. albicans* but only 37.2% among the *C. parapsilosis* complex (95% CI 27.3 to 48.3). The current EUCAST tables add a layer of interpretation to the fluconazole contrasts. For *N. glabratus*, the entire wild-type population falls within the susceptible, increased exposure category and isolates are interpreted as resistant only above 16 mg/L [22], so the 81.7% reported as susceptible by the commercial system describes something categorically different from an EUCAST susceptibility rate. For *P. kudriavzevii*, EUCAST sets no fluconazole breakpoints, states that testing is not recommended because the species is intrinsically resistant, and permits isolates to be reported as resistant without testing [22].

The complete absence of fluconazole-susceptible classifications among *P. kudriavzevii* across all four intervals is fully concordant with the intrinsic fluconazole resistance of this species; in that respect the commercial system and the established biology agree perfectly. We nevertheless present this result as a descriptive anchor rather than as a control, for three reasons. First, the fluconazole epidemiological cut-off for this species is 128 mg/L [30], above the single 64 µg/mL concentration present on the panel, so the observation is neither a clean expression of the biology nor a demonstration that the assay behaved correctly. Second, an invariant phenotype is sensitive to false-susceptible drift, and its constancy over eight years is reassuring in that respect, but it is insensitive to the graded, agent-specific shifts described in Section 3.7, because a value fixed at zero cannot register them. Third, it says nothing about whether the species label was correctly applied, which the identification performance documented in Section 4.1 leaves open.

The same caution applies to the other species-specific contrasts. Because non-albicans identification by this system is unreliable, isolates grouped as *P. kudriavzevii* and *C. parapsilosis* complex are less securely assigned than the *C. albicans* group. The phenotypic contrasts are real within the commercial-system labels; their attribution to a named species requires confirmation by MALDI-TOF with an updated database or by sequencing, which EUCAST identifies as a precondition for interpreting susceptibility results where categorical assignment is uncertain [34]. This does not weaken the practical conclusion that species-level identification and species-specific reporting are necessary whenever susceptibility testing is undertaken for vaginal yeast isolates, but it means that the nystatin signal in the *C. parapsilosis* complex should be treated as a hypothesis for external validation in cohorts with definitive species identification, not as a property of that species established here.

### 4.5. Temporal Signals in This Dataset Do Not Constitute Evidence of Resistance Evolution

The post hoc consistency analyses in Section 3.7 impose an important constraint on how the temporal results should be read, and we regard this as the most consequential methodological message of the study. Two patterns are difficult to reconcile with biological change: the simultaneous final-period rise in four pharmacologically distinct agents in *C. albicans*, absent from non-albicans isolates tested on the same panel, and the synchronous decline of clotrimazole classifications across three non-albicans species during 2020 to 2022, with complete recovery afterwards and no parallel movement in econazole. Resistance dynamics in a community-derived, largely outpatient population do not plausibly generate coordinated two-year reversals of this magnitude in several species at once, restricted to a single agent.

Analytical or process explanations are more parsimonious, and two are documented for this laboratory (Section 2.3). The change in personnel performing and reading the tests during 2022 to 2024 coincides with the *C. albicans* miconazole and nystatin trough that immediately preceded the final-period rise, and endpoint interpretation in a colorimetric system is precisely the step that a change in operator would be expected to affect. It does not account for the clotrimazole anomaly of 2020 to 2022. The laboratory’s quality records constrain the alternatives (Section 3.7): every lot passed acceptance testing with a wild-type reference strain, which excludes a gross lot failure, and lots turned over several times a year, so a two-year anomaly cannot reflect a single lot. What the records cannot exclude is precisely the mechanism proposed below, a displacement of the effective endpoint by a fraction of a dilution, which categorical acceptance testing with a single strain would not register, or a change in how ambiguous colour changes were read. On this basis the personnel change remains the most specific explanation for the 2022 to 2024 trough, whereas the agent-restricted clotrimazole anomaly would require either a sustained effect across a series of lots on the clotrimazole well or a reading effect specific to that well, neither of which can be tested retrospectively. This is why the temporal patterns are reported as compatible with analytical variation rather than attributed to it.

The position of each well relative to the MIC distribution of the organism provides a mechanism that accounts for which agents varied and which did not. For *C. albicans*, fluconazole is tested at 64 µg/mL, far above the EUCAST wild-type range and breakpoints, so the well functions as a screen for high-level resistance and is insensitive to small changes in reading or reagent performance. The remaining four agents are tested at 1 to 2 µg/mL, within or close to the range of MICs reported for *Candida* against topical imidazoles and polyenes [3,4]. A single well positioned within a susceptibility distribution bisects that distribution, so a small displacement of the effective endpoint, whether from a lot difference or from a different reader interpreting an ambiguous orange, reclassifies a substantial proportion of isolates. Supplementary Analysis S1 quantifies this with a simple model of log-normally distributed MICs: when the well lies within one doubling dilution above the modal MIC, so that roughly 65 to 90% of isolates are classified as susceptible, a displacement of the effective endpoint by only half a dilution changes the proportion classified by about 7 to 25 percentage points depending on the width of the distribution, a range that encompasses the observed between-period changes for the four topical agents in *C. albicans* (5 to 15 points), whereas the same displacement at a well six or more dilutions above the mode, the fluconazole configuration, changes the proportion by less than 0.01 points (Supplementary Figure S1). The four agents tested near the distribution are precisely the four that showed statistically robust temporal change in *C. albicans*, and fluconazole, tested far above it, was the only agent that remained stable. The same reasoning may account for the *C. parapsilosis* complex nystatin phenotype, where an aggregate of 37.2% oscillated between 18.2% and 50.0% in a pattern characteristic of a threshold cutting through a distribution. This account is a mechanism consistent with the data rather than a demonstration, because no epidemiological cut-off values exist for the four topical agents and the position of each well relative to the wild-type distribution is inferred from published MIC ranges.

Several non-analytical explanations remain uncontrolled. Referral patterns and case-mix may have shifted over eight years, local antifungal prescribing may have changed, and the second interval coincides with the COVID-19 pandemic, during which access to gynecological care and thresholds for sampling plausibly changed. A related statistical point is that significant differences among periods do not indicate directional evolution: temporal heterogeneity, meaning that proportions differ among individual periods, must be distinguished from directional change, meaning a sustained progressive increase or decrease, and both must be distinguished from measurement instability. Multiplicity correction protects against chance findings but offers no protection against systematic measurement shifts. We therefore do not interpret the four statistically robust *C. albicans* trends as evidence of improving susceptibility in the population, and we advance no claim of temporal change: the appropriate reading is that categorical output from a single commercial system, aggregated over eight years, carries a component of analytical variation that formal statistical significance does not remove.

### 4.6. Cohort Structure Imposes a Second Constraint on Temporal Inference

The unique-patient principle, adopted to prevent repeated isolates from dominating prevalence estimates, has a directional consequence for longitudinal analysis. Because each woman contributes only her first isolate over the whole eight years, later intervals are composed exclusively of women not previously represented in the cohort. Women with recurrent or persistent disease, who are more likely to have had repeated antifungal exposure and are plausibly enriched for reduced susceptibility, are assigned to the earliest interval in which they appear, whereas later periods are progressively enriched with newly included women who may have been less heavily pre-treated, although no treatment data were available to test this. This structural feature could produce rising proportions classified as susceptible over time independently of any change in the organisms or in the assay, and it therefore provides a second non-biological explanation for the *C. albicans* trends. It is a central limitation of the temporal analysis rather than a technical footnote, and it is stated as such in the Abstract and Conclusions. A sensitivity analysis retaining all isolates per patient, with correction for within-patient clustering, would quantify its direction and magnitude; it could not be performed here because the surveillance dataset was compiled in aggregated form from the outset and isolate-level records with collection dates were not retained (Section 5).

### 4.7. Translational Implications and Future Directions

The surveillance dataset provides an epidemiologically grounded basis for prospective work. A representative panel should include *C. albicans* isolates with contrasting phenotypic profiles, *N. glabratus*, *P. kudriavzevii* as a fluconazole-resistant comparator, and *C. parapsilosis* complex isolates, particularly those associated with the nystatin signal identified here. Prospective testing should combine standardized MIC determination by EUCAST methodology with definitive species identification, and should extend to fungicidal activity, biofilm inhibition and testing at physiologically relevant vaginal pH, since standardized in vitro conditions do not reproduce the mucosal microenvironment in which topical agents act. Candidate topical adjuncts should be assessed jointly for efficacy, vaginal epithelial cytotoxicity and effects on protective lactobacilli, rather than for activity in isolation.

One avenue that would otherwise be decisive is closed for this dataset. Retesting stored isolates from different intervals by a reference method under contemporaneous quality control would determine directly whether the temporal patterns reflect the organisms or the measurement, but the isolates were discarded after testing in accordance with national regulations on medical waste [28], as is standard practice in diagnostic laboratories without a research biobank. The caution advised in Section 4.5 and Section 4.6 is therefore permanent rather than provisional, which is itself an argument for prospective biobanking wherever long-term surveillance is intended, and for qualifying the in-house performance of any commercial system at the outset. A defined protocol for this already exists: running the EUCAST quality control strains repeatedly, followed by the confirmatory panel of unrelated clinical isolates, would establish whether a system can be used with EUCAST breakpoints at all and would resolve in a single experiment the questions that eight years of retrospective categorical data cannot answer [29]. We would encourage any laboratory maintaining long-term surveillance on a commercial antifungal panel to perform this qualification before reporting temporal trends.

The study laboratory has since moved to quantitative MIC determination by EUCAST reference methodology, with susceptibility testing of vaginal isolates confined to fluconazole at clinical request or in refractory candidiasis, and full characterization reserved for invasive disease and immunosuppressed patients. That is the correct direction for interpretable and comparable results, but it also means that routine categorical susceptibility data for topical antifungals in VVC will not be produced again, while topical imidazoles and nystatin remain first-line therapy for a common condition with no recognized laboratory method for assessing susceptibility to them. That evidence gap is a standard-setting problem rather than a local one, and it will not be closed by individual laboratories.

## 5. Strengths and Limitations

The principal strengths of this study are the size and duration of the dataset, 2789 isolates from 2789 unique patients over eight consecutive years; methodological continuity, with a single system used according to the manufacturer’s instructions throughout; species-specific analysis, which avoids masking differences within pooled data; and an analytical strategy that used exact testing where expected counts were small, reported confidence intervals for all proportions, controlled multiplicity, and tested the temporal signals against the patterns expected under competing explanations rather than accepting them at face value.

Several limitations must be acknowledged. First, the study was retrospective and laboratory based. Symptom status, clinical diagnostic criteria, previous treatment and recurrence status were not recorded, and fungal culture was most often requested as part of a panel rather than for a specific suspicion of candidiasis (Section 2.2), so the cohort represents women from whom *Candida* was recovered and includes an unknown proportion of asymptomatic colonization; it may therefore not be representative of symptomatic VVC, which limits clinical interpretation. Second, the study was conducted at a single hospital in north-western Romania; this is a strength for longitudinal consistency, but the findings may not apply to other populations or healthcare settings. Third, the number of cultures performed within each interval and the underlying population denominators were unavailable, so the increase in absolute isolate numbers cannot be interpreted as an increase in incidence or prevalence. Fourth, susceptibility was determined by a commercial colorimetric system rather than a quantitative reference method, and the classifications must not be treated as equivalent to EUCAST MIC-based categories. Fifth, and most importantly for the temporal analyses, the laboratory quality records consist of categorical acceptance testing of each lot with a single reference strain, which recorded no deviation, and lot-level records retrieved for 2024 and 2025 (Supplementary Table S5); reading logs identifying operators and quantitative control results are not available, so the analytical explanations advanced in Section 4.5 cannot be resolved from the dataset alone. Sixth, the unique-patient design progressively enriches later intervals with newly included women and biases temporal comparisons in the direction observed (Section 4.6); because the dataset was compiled in aggregated form and isolate-level records were not retained, a sensitivity analysis retaining all isolates per patient was not possible. Seventh, the system does not reliably separate *C. albicans* from *C. dubliniensis* [27], nor the members of the *C. parapsilosis* complex, so these categories should be understood as species complexes. Eighth, the smaller numbers of non-albicans isolates, particularly the *C. parapsilosis* complex, limit the precision of longitudinal analyses; several strata contained fewer than ten isolates per interval.

These limitations define the scope of the study as longitudinal laboratory surveillance of commercial-system species assignments and antifungal phenotypes, rather than an analysis of clinical therapeutic efficacy or of reference-method-defined resistance prevalence.

## 6. Conclusions

Across 2789 vaginal *Candida* isolates from 2789 unique patients, the distribution of species assignments generated by Integral System YEASTS Plus remained statistically stable over eight years, with no shift toward non-albicans species. This is a conclusion about the assignments, not about vaginal *Candida* ecology: the documented identification performance of the system implies that the *C. albicans* fraction is overestimated and that the true non-albicans fraction is higher.

Antifungal phenotypes differed markedly between species. *P. kudriavzevii* was never classified as fluconazole-susceptible, in agreement with its intrinsic resistance, and only 37.2% of *C. parapsilosis* complex isolates were classified as nystatin-susceptible; pooled genus-level reporting would have concealed both. These contrasts are the most robust findings of the study and support species-level identification and species-specific reporting for vaginal yeast isolates, with the nystatin signal now requiring external validation in cohorts with definitive species identification. *C. albicans* fluconazole classifications remained consistently high, which is a statement about the stability of the measurement rather than about resistance prevalence, which the single 64 µg/mL well cannot resolve.

Directional temporal changes surviving multiplicity correction were confined to *C. albicans*, but their structure, a synchronous final-period rise across pharmacologically distinct agents together with a simultaneous decline and recovery of clotrimazole classifications in three non-albicans species, is more consistent with analytical variation than with resistance evolution, and the first-isolate design independently biases temporal comparisons in the same direction. We therefore advance no claim of temporal change in susceptibility. The wider message is methodological: categorical output from a commercial system should not be equated with reference MIC-based categories, temporal signals in retrospective surveillance must be tested against non-biological explanations before they are reported, and laboratories maintaining long-term surveillance on commercial panels should qualify the system against the reference method and biobank isolates at the outset.

## Figures and Tables

**Table 1:** **Species distribution by surveillance period and test for proportional temporal trend.**

Species	2018–2020 *n* (%)	2020–2022 *n* (%)	2022–2024 *n* (%)	2024–2026 *n* (%)	*p* Trend	*q* Trend
*C. albicans*	445 (86.9)	456 (81.1)	632 (84.4)	826 (85.5)	0.835	0.985
*N. glabratus* (*C. glabrata*)	41 (8.0)	58 (10.3)	64 (8.5)	72 (7.5)	0.333	0.648
*P. kudriavzevii* (*C. krusei*)	16 (3.1)	27 (4.8)	29 (3.9)	35 (3.6)	0.985	0.985
*C. parapsilosis* complex	9 (1.8)	19 (3.4)	22 (2.9)	28 (2.9)	0.389	0.648
Other *Candida* spp.	1 (0.2)	2 (0.4)	2 (0.3)	5 (0.5)	0.363	0.648
Total	512 (100)	562 (100)	749 (100)	966 (100)		

Percentages are column percentages within each surveillance period. *p* trend derives from a binomial generalized linear model with period entered as an ordinal predictor; *q* trend is the Benjamini–Hochberg-adjusted value across the five species. Overall distribution: chi-square = 10.97, df = 12, asymptotic *p* = 0.532; Monte Carlo conditional *p* = 0.532; four principal species only, chi-square = 9.72, df = 9, *p* = 0.373.

**Table 2:** **Aggregate proportions classified as susceptible by Integral System YEASTS Plus, by species and antifungal agent.**

Species	Clotrimazole	Econazole	Miconazole	Fluconazole	Nystatin
*C. albicans (n = 2359)*	81.7 (80.1–83.2)	83.2 (81.7–84.7)	91.6 (90.5–92.7)	97.3 (96.6–97.9)	89.8 (88.5–90.9)
*N. glabratus* (*C. glabrata*) (*n* = 235)	80.9 (75.3–85.4)	75.7 (69.9–80.8)	81.3 (75.8–85.7)	81.7 (76.3–86.1)	82.1 (76.7–86.5)
*P. kudriavzevii* (*C. krusei*) (*n* = 107)	77.6 (68.8–84.4)	89.7 (82.5–94.2)	58.9 (49.4–67.7)	**0.0** **(0.0–3.5)**	74.8 (65.8–82.0)
*C. parapsilosis* complex (*n* = 78)	69.2 (58.3–78.4)	66.7 (55.6–76.1)	75.6 (65.1–83.8)	79.5 (69.2–87.0)	**37.2** **(27.3–48.3)**

Values are percentages classified as susceptible, with Wilson 95% confidence intervals in parentheses. These are commercial-system classifications and must not be read as EUCAST MIC-based susceptibility rates. Species names are commercial-system assignments; legacy names generated by the system are given in parentheses. Values in bold are the two species-restricted phenotypes discussed in Section 4.4: the invariant fluconazole classification of *P. kudriavzevii*, consistent with its intrinsic resistance, and the low nystatin classification of the *C. parapsilosis* complex.

**Table 3:** **Temporal distribution of isolates classified as susceptible, with Monte Carlo conditional and multiplicity-adjusted testing.**

Species and Agent	2018–2020 *n/N* (%)	2020–2022 *n/N* (%)	2022–2024 *n/N* (%)	2024–2026 *n/N* (%)	*p* Glob	*q* Glob	*p* Trend	*q* Trend
*C. albicans*								
Clotrimazole	348/445 (78.2)	348/456 (76.3)	506/632 (80.1)	726/826 (87.9)	<0.001	<0.001	<0.001	<0.001
Econazole	352/445 (79.1)	375/456 (82.2)	509/632 (80.5)	727/826 (88.0)	<0.001	<0.001	<0.001	<0.001
Miconazole	399/445 (89.7)	411/456 (90.1)	539/632 (85.3)	813/826 (98.4)	<0.001	<0.001	<0.001	<0.001
Fluconazole	433/445 (97.3)	438/456 (96.1)	612/632 (96.8)	812/826 (98.3)	0.094	0.199	0.117	0.278
Nystatin	389/445 (87.4)	399/456 (87.5)	523/632 (82.8)	807/826 (97.7)	<0.001	<0.001	<0.001	<0.001
*N. glabratus* (*C. glabrata*)								
Clotrimazole	35/41 (85.4)	35/58 (60.3)	54/64 (84.4)	66/72 (91.7)	<0.001	<0.001	0.016	0.059
Econazole	32/41 (78.0)	45/58 (77.6)	49/64 (76.6)	52/72 (72.2)	0.874	0.911	0.440	0.612
Miconazole	29/41 (70.7)	47/58 (81.0)	52/64 (81.3)	63/72 (87.5)	0.186	0.321	0.042	0.133
Fluconazole	32/41 (78.0)	47/58 (81.0)	53/64 (82.8)	60/72 (83.3)	0.911	0.911	0.477	0.612
Nystatin	33/41 (80.5)	48/58 (82.8)	51/64 (79.7)	61/72 (84.7)	0.884	0.911	0.654	0.690
*P. kudriavzevii* (*C. krusei*)								
Clotrimazole	14/16 (87.5)	14/27 (51.9)	23/29 (79.3)	32/35 (91.4)	0.001	0.004	0.064	0.174
Econazole	15/16 (93.8)	22/27 (81.5)	24/29 (82.8)	35/35 (100.0)	0.045	0.106	0.182	0.384
Miconazole	10/16 (62.5)	17/27 (63.0)	17/29 (58.6)	19/35 (54.3)	0.911	0.911	0.475	0.612
Fluconazole	0/16 (0.0)	0/27 (0.0)	0/29 (0.0)	0/35 (0.0)	n.a.	n.a.	n.a.	n.a.
Nystatin	12/16 (75.0)	21/27 (77.8)	23/29 (79.3)	24/35 (68.6)	0.767	0.911	0.522	0.620
*C. parapsilosis* complex								
Clotrimazole	8/9 (88.9)	8/19 (42.1)	17/22 (77.3)	21/28 (75.0)	0.023	0.062	0.441	0.612
Econazole	6/9 (66.7)	14/19 (73.7)	15/22 (68.2)	17/28 (60.7)	0.854	0.911	0.483	0.612
Miconazole	6/9 (66.7)	15/19 (78.9)	18/22 (81.8)	20/28 (71.4)	0.768	0.911	0.961	0.961
Fluconazole	8/9 (88.9)	16/19 (84.2)	17/22 (77.3)	21/28 (75.0)	0.763	0.911	0.297	0.564
Nystatin	4/9 (44.4)	7/19 (36.8)	4/22 (18.2)	14/28 (50.0)	0.135	0.257	0.592	0.662

Percentages are isolates classified as susceptible by Integral System YEASTS Plus. p glob is a Monte Carlo approximation to a conditional exact test for the 2 × 4 table with fixed margins (200,000 replicates), used in preference to the asymptotic chi-square because expected counts fell as low as 1.6 in non-albicans strata. *p* trend derives from a binomial generalized linear model with period as an ordinal predictor. *q* values are Benjamini–Hochberg-adjusted within each family of 19 testable comparisons. n.a., not applicable: all 107 *P. kudriavzevii* isolates were tested for fluconazole and none was classified as susceptible in any interval, so the phenotype was invariant and inferential testing had no variance to evaluate. Species names are commercial-system assignments; legacy names are given in parentheses.

## Data Availability

The aggregated data supporting the findings of this study are presented in full in Supplementary Table S1 and are available from the corresponding author upon reasonable request, subject to applicable institutional and ethical requirements. Isolate-level records, including collection dates and repeated determinations from individual patients, were not retained in retrievable form and cannot be made available. The full analysis code used to generate all tests, adjusted *p* values and figures is provided in the Supplementary Materials, together with a completed STROBE checklist.

## Supplementary Materials

The following supporting information can be downloaded at: https://www.mdpi.com/article/10.3390/jof12100756/s1. Table S1: complete susceptible and non-susceptible counts by species, antifungal agent and surveillance period; Table S2: temporal trend models with odds ratios and 95% confidence intervals; Table S3: STROBE checklist; Table S4: sensitivity of the Monte Carlo conditional tests to the number of replicates; Supplementary Analysis S1: Sensitivity of a single-concentration categorical endpoint to small displacements; Figure S1: Sensitivity of a single-concentration categorical endpoint to displacement; Table S5: kit lots and control-strain acceptance testing for 2024 and 2025; Supplementary analysis code with dependency versions.
