# Supplementary material for: Evolution of Vaginal Candida spp. over Eight Years: Species Stability and Variability in Antifungal Susceptibility Classifications Using the Integral System YEASTS Plus
Source: J Fungi (Basel). 2026 Oct 9;12(10):756. doi: 10.3390/jof12100756 (PMC13706058; doi:10.3390/jof12100756)
Supplement: Supplementary file 1 [file jof-12-00756-s001.zip › Supplementary_Material_REVISED.pdf]

Supplementary Material

Evolution of Vaginal *Candida* spp. over Eight Years: Species Stability and Variability in Antifungal Susceptibility Classifications Using the Integral System YEASTS Plus

Supplementary Table S1

Table S1. Complete susceptible and non-susceptible counts by species, antifungal agent and surveillance period. Values are the raw counts from which every percentage, test and model in the main manuscript is derived.

Species	Agent	2018-2020<br>S / NS	2020-2022<br>S / NS	2022-2024<br>S / NS	2024-2026<br>S / NS
C. albicans (n=2,359)	Clotrimazole	348 / 97	348 / 108	506 / 126	726 / 100
	Econazole	352 / 93	375 / 81	509 / 123	727 / 99
	Miconazole	399 / 46	411 / 45	539 / 93	813 / 13
	Fluconazole	433 / 12	438 / 18	612 / 20	812 / 14
	Nystatin	389 / 56	399 / 57	523 / 109	807 / 19
N. glabratus (*C. glabrata*) (n=235)	Clotrimazole	35 / 6	35 / 23	54 / 10	66 / 6
	Econazole	32 / 9	45 / 13	49 / 15	52 / 20
	Miconazole	29 / 12	47 / 11	52 / 12	63 / 9
	Fluconazole	32 / 9	47 / 11	53 / 11	60 / 12
	Nystatin	33 / 8	48 / 10	51 / 13	61 / 11
P. kudriavzevii (*C. krusei*) (n=107)	Clotrimazole	14 / 2	14 / 13	23 / 6	32 / 3
	Econazole	15 / 1	22 / 5	24 / 5	35 / 0
	Miconazole	10 / 6	17 / 10	17 / 12	19 / 16
	Fluconazole	0 / 16	0 / 27	0 / 29	0 / 35
	Nystatin	12 / 4	21 / 6	23 / 6	24 / 11
C. parapsilosis complex (n=78)	Clotrimazole	8 / 1	8 / 11	17 / 5	21 / 7
	Econazole	6 / 3	14 / 5	15 / 7	17 / 11
	Miconazole	6 / 3	15 / 4	18 / 4	20 / 8
	Fluconazole	8 / 1	16 / 3	17 / 5	21 / 7
	Nystatin	4 / 5	7 / 12	4 / 18	14 / 14

S, classified as susceptible by Integral System YEASTS Plus; NS, not classified as susceptible. Ten isolates assigned to other Candida spp. (1, 2, 2 and 5 across the four periods) were included in the species-distribution analysis but not in susceptibility analyses. Classifications are commercial-system outputs and are not equivalent to EUCAST MIC-based categories.

Supplementary Table S2

Table S2. Temporal trend models with odds ratios and 95% confidence intervals. Estimated from binomial generalized linear models with a logit link and surveillance period entered as an ordinal predictor (1 to 4).

Species	Agent	OR per period (95% CI)	p	q	Interpretation
C. albicans	Clotrimazole	1.27 (1.16 to 1.39)	<0.001	<0.001	robust
	Econazole	1.21 (1.10 to 1.33)	<0.001	<0.001	robust
	Miconazole	1.42 (1.24 to 1.61)	<0.001	<0.001	robust
	Fluconazole	1.19 (0.96 to 1.48)	0.117	0.278	not significant
	Nystatin	1.42 (1.26 to 1.60)	<0.001	<0.001	robust
N. glabratus (*C. glabrata*)	Clotrimazole	1.46 (1.07 to 1.97)	0.016	0.059	exploratory
	Econazole	0.90 (0.68 to 1.18)	0.440	0.612	not significant
	Miconazole	1.37 (1.01 to 1.86)	0.042	0.133	exploratory
	Fluconazole	1.12 (0.82 to 1.51)	0.477	0.612	not significant
	Nystatin	1.07 (0.79 to 1.46)	0.654	0.690	not significant
P. kudriavzevii (*C. krusei*)	Clotrimazole	1.51 (0.98 to 2.32)	0.064	0.174	not significant
	Econazole	1.49 (0.83 to 2.69)	0.182	0.384	not significant
	Miconazole	0.88 (0.61 to 1.26)	0.475	0.612	not significant
	Fluconazole	not estimable	n.a.	n.a.	invariant
	Nystatin	0.87 (0.57 to 1.32)	0.522	0.620	not significant
C. parapsilosis complex	Clotrimazole	1.20 (0.75 to 1.92)	0.441	0.612	not significant
	Econazole	0.85 (0.53 to 1.35)	0.483	0.612	not significant
	Miconazole	0.99 (0.60 to 1.64)	0.961	0.961	not significant
	Fluconazole	0.74 (0.42 to 1.31)	0.297	0.564	not significant
	Nystatin	1.13 (0.72 to 1.78)	0.592	0.662	not significant

OR, odds ratio for being classified as susceptible per one-period increment. q, Benjamini-Hochberg adjusted p value across the family of 19 testable trend comparisons. Robust, q < 0.05; exploratory, nominally significant but q >= 0.05; not estimable, phenotype invariant across all periods (P. kudriavzevii and fluconazole, 0/107). Odds ratios describe change in commercial-system classification over calendar time and must not be read as measures of biological resistance evolution; see Sections 3.7 and 4.5 of the main manuscript.

Supplementary Table S3. STROBE checklist

Reporting checklist for observational studies, cross-sectional and cohort items as applicable to a retrospective laboratory-based surveillance study.

Item	Recommendation	Where addressed	Page
1a	Study design indicated in title or abstract	Title; Abstract, Materials and Methods	1, 3
1b	Informative and balanced abstract	Single-paragraph abstract (Journal of Fungi format, without headings) covering background, objectives, methods, results and conclusions	1
2	Scientific background and rationale	Introduction	2–3
3	Specific objectives, including any prespecified hypotheses	Introduction, final paragraph (four stated objectives: species distribution, species-specific and temporal phenotypes, internal consistency of temporal signals, and identification of signals for prospective reference-method testing). The internal-consistency analyses reported in Sections 3.7 and 4.6 were not prespecified and are reported as post hoc and exploratory (Section 2.6)	2–3
4	Study design, key elements	Section 2.1	3
5	Setting, locations, periods of recruitment and data collection	Section 2.1 (Pelican Hospital, Oradea; August 2018 to July 2026)	3
6a	Eligibility criteria, sources and selection methods	Section 2.2 (microbiological eligibility; first chronologically eligible isolate per unique patient, matched by laboratory identifier)	3
6b	Matching criteria and number of exposed and unexposed	Not applicable: single-cohort descriptive surveillance without exposure groups	—
7	Variables defined, including outcomes and effect modifiers	Sections 2.3 and 2.4	4–5
8	Data sources and measurement methods for each variable	Sections 2.3 and 2.4 (Integral System YEASTS Plus)	4–5
9	Efforts to address potential sources of bias	Sections 2.2, 2.6, 3.7, 4.5, 4.6 and 5	3, 6, 11–12, 16–17, 18–19
10	Study size explained	Section 2.2 (all eligible isolates in the period; no a priori sample size)	3
11	Handling of quantitative variables and groupings	Section 2.4 (categorization as susceptible versus non-susceptible); Section 2.5 (period as ordinal predictor)	5
12a	Statistical methods, including confounding control	Section 2.5	5–6
12b	Methods for subgroup and interaction analyses	Section 2.5 (species-stratified analyses)	5–6
12c	Handling of missing data	Section 5 (patient-level variables, period-specific denominators, laboratory quality records and isolate-level records absent from the aggregated dataset)	18–19
12d	Handling of loss to follow-up	Not applicable: cross-sectional laboratory surveillance without follow-up	—
12e	Sensitivity analyses	Sections 2.6 and 3.7 (post hoc internal consistency analyses); Supplementary Table S4 (Monte Carlo replicate sensitivity); Supplementary Analysis S1 (endpoint displacement model)	6, 11–12
13a	Participants at each stage	Section 2.2 and Section 3.1 (17,267 vaginal fungal cultures performed; 2,789 isolates from 2,789 unique patients retained)	3, 7
13b	Reasons for non-participation	Section 2.2 (deduplication to one isolate per patient); no other exclusions applied	3
13c	Flow diagram	Not provided; participant flow is described in full in Section 2.2	3
14a	Descriptive data	Section 3.1 and Table 1; Figure 1	7–8
14b	Participants with missing data for each variable	Not applicable: all included isolates had complete species and susceptibility classification (Section 2.4)	—
14c	Follow-up time	Not applicable	—
15	Outcome data	Tables 2 and 3; Supplementary Table S1	8–9
16a	Main results with precision estimates	Tables 2 and 3; Supplementary Table S2 (Wilson 95% CI; odds ratios with 95% CI)	8–9
16b	Category boundaries for continuous variables	Not applicable: no continuous variables were categorized; surveillance period was analysed as an ordinal predictor (Section 2.5)	—
16c	Translation of relative risk into absolute risk	Not applicable: no risk estimates were derived	—

Item	Recommendation	Where addressed	Page
17	Other analyses performed	Section 3.7 and Figure 4 (post hoc exploratory internal-consistency analyses); Section 3.2 (restricted four-species analysis)	11–12
18	Key results summarised with reference to objectives	Section 6	19
19	Limitations, including sources of bias and imprecision	Section 5	18–19
20	Cautious overall interpretation	Sections 4.5, 4.6 and 6	16–17, 19
21	Generalisability	Sections 4.1 and 5	12–13, 18–19
22	Funding and role of funders	Funding statement	20

Page numbers refer to the revised manuscript file (clean version).

Supplementary analysis code

The complete Python code used to generate every statistical test, multiplicity adjustment and figure reported in this manuscript is provided as a separate file (Supplementary_analysis_code.py). It reproduces the species-distribution tests, the Monte Carlo conditional tests with fixed margins for all species-agent tables, the binomial generalized linear models with the odds ratios and 95% confidence intervals reported in Table S2, the Benjamini-Hochberg adjustments, the Wilson confidence intervals, all four main-text figures, the replicate sensitivity analysis of Table S4 and the endpoint displacement model of Supplementary Analysis S1, from the counts given in Supplementary Table S1. The random seed (20260822) is fixed and the exact versions of all dependencies are listed in the header of the code file (Python 3.12.3, NumPy 2.4.4, SciPy 1.17.1, statsmodels 0.14.6, Matplotlib 3.10.8), so all reported values are exactly reproducible.

Supplementary Table S4

Table S4. Sensitivity of the Monte Carlo conditional tests to the number of replicates. p values for the 2 × 4 species-agent tables obtained with 50,000, 100,000, 200,000 (reported in the main text, Table 3) and 1,000,000 replicates, all with the same fixed random seed.

Species	Agent	50,000	100,000	200,000	1,000,000
C. albicans	Clotrimazole	<0.001	<0.001	<0.001	<0.001
	Econazole	<0.001	<0.001	<0.001	<0.001
	Miconazole	<0.001	<0.001	<0.001	<0.001
	Fluconazole	0.097	0.095	0.094	0.094
	Nystatin	<0.001	<0.001	<0.001	<0.001
N. glabratus	Clotrimazole	<0.001	<0.001	<0.001	<0.001
	Econazole	0.876	0.876	0.874	0.875
	Miconazole	0.184	0.185	0.186	0.185
	Fluconazole	0.911	0.910	0.911	0.912
	Nystatin	0.883	0.883	0.884	0.883
P. kudriavzevii	Clotrimazole	0.001	0.001	0.001	0.002
	Econazole	0.045	0.044	0.045	0.045
	Miconazole	0.909	0.910	0.911	0.910
	Fluconazole	n.a.	n.a.	n.a.	n.a.
	Nystatin	0.767	0.768	0.767	0.766
C. parapsilosis complex	Clotrimazole	0.022	0.024	0.023	0.023
	Econazole	0.855	0.851	0.854	0.854
	Miconazole	0.767	0.768	0.768	0.769

Species	Agent	50,000	100,000	200,000	1,000,000
	Fluconazole	0.758	0.762	0.763	0.761
	Nystatin	0.136	0.134	0.135	0.134

Values are Monte Carlo approximations to the conditional exact test with fixed margins, using the Pearson chi-square statistic and the NumPy default random generator with seed 20260822. The 200,000-replicate column reproduces the p glob values reported in Table 3 of the main manuscript. Across the 19 testable comparisons, no p value differed by more than 0.005 between replicate counts, and neither nominal significance ($p < 0.05$) nor Benjamini-Hochberg adjusted significance ($q < 0.05$) changed for any comparison. n.a., not applicable: invariant phenotype (*P. kudriavzevii* and fluconazole, 0/107).

Supplementary Analysis S1. Sensitivity of a single-concentration categorical endpoint to small displacements

Rationale. Integral System YEASTS Plus presents each antifungal in a single well at one fixed concentration, so the proportion of isolates classified as susceptible equals the proportion whose minimum inhibitory concentration (MIC) lies at or below that concentration. Any factor that displaces the effective endpoint by a fraction of a doubling dilution, such as lot-to-lot variation in the amount of drug in the well or a different operator reading an ambiguous orange colour, will reclassify the isolates whose MICs lie close to the well concentration. The magnitude of the effect therefore depends on where the well concentration sits relative to the MIC distribution of the organism.

Model. MICs on the log2 scale were modelled as normally distributed with standard deviation σ doubling dilutions (σ = 0.75, 1.0 and 1.25, spanning the width typical of wild-type MIC distributions) and mode at zero. The well concentration was placed d doubling dilutions above the mode (d = 0.5 to 6). The proportion classified as susceptible is $\Phi(d/\sigma)$, where Φ is the standard normal cumulative distribution function, and after a displacement of the effective endpoint by δ dilutions it becomes $\Phi((d + \delta)/\sigma)$. The change in the proportion classified was computed for δ = ±0.5 and ±1 dilution. No MIC data from the present isolates enter the model, which is intentionally generic; it is anchored to the study only through the position of the fluconazole well, 64 μg/mL, which lies seven doubling dilutions above the EUCAST wild-type epidemiological cut-off of 0.5 mg/L for *C. albicans*, and through the observation that the four topical agents are tested at 1 to 2 μg/mL, within or close to the range of MICs reported for *Candida* against topical imidazoles and polyenes (main text, references 3 and 4).

Result. When the well lies within one doubling dilution above the modal MIC, so that roughly 65 to 90% of isolates are classified as susceptible (the situation of the four topical agents in *C. albicans*), a displacement of only half a dilution changes the proportion classified by about 7 to 25 percentage points depending on σ (Table below and Figure S1). This range encompasses the between-period changes observed for clotrimazole, econazole, miconazole and nystatin in *C. albicans* (5 to 15 percentage points; Table 3 of the main text). At six or more dilutions above the mode, the configuration of the fluconazole well for *C. albicans*, the same displacement changes the proportion by less than 0.01 percentage points. A single-concentration endpoint is therefore highly sensitive to small analytical displacements precisely for the agents whose wells fall within the MIC distribution, and insensitive for an agent tested far above it, which is the pattern observed in Section 3.7 of the main text.

Table. Change in the proportion classified as susceptible after displacement of the effective endpoint. Entries are percentage points; S0 is the proportion classified before displacement.

σ	d (dilutions)	S0 (%)	δ = −0.5	δ = +0.5	δ = −1	δ = +1
0.75	0.5	74.8	-24.8	+16.1	-49.5	+23.0
	1	90.9	-16.1	+6.8	-40.9	+8.7
	1.5	97.7	-6.8	+1.9	-23.0	+2.2
	2	99.6	-1.9	+0.3	-8.7	+0.4
	3	100.0	-0.0	+0.0	-0.4	+0.0
	6	100.0	-0.0	+0.0	-0.0	+0.0
1.00	0.5	69.1	-19.1	+15.0	-38.3	+24.2

σ	d (dilutions)	S0 (%)	δ = −0.5	δ = +0.5	δ = −1	δ = +1
	1	84.1	-15.0	+9.2	-34.1	+13.6
	1.5	93.3	-9.2	+4.4	-24.2	+6.1
	2	97.7	-4.4	+1.7	-13.6	+2.1
	3	99.9	-0.5	+0.1	-2.1	+0.1
	6	100.0	-0.0	+0.0	-0.0	+0.0
1.25	0.5	65.5	-15.5	+13.3	-31.1	+23.0
	1	78.8	-13.3	+9.7	-28.8	+15.7
	1.5	88.5	-9.7	+6.0	-23.0	+9.2
	2	94.5	-6.0	+3.2	-15.7	+4.7
	3	99.2	-1.5	+0.6	-4.7	+0.8
	6	100.0	-0.0	+0.0	-0.0	+0.0

σ, standard deviation of the log2 MIC distribution in doubling dilutions; d, distance of the well concentration above the modal MIC; δ, displacement of the effective endpoint in doubling dilutions (positive values correspond to a higher effective concentration or a more lenient reading, negative values to the reverse). Values of 0.0 denote changes below 0.05 percentage points.

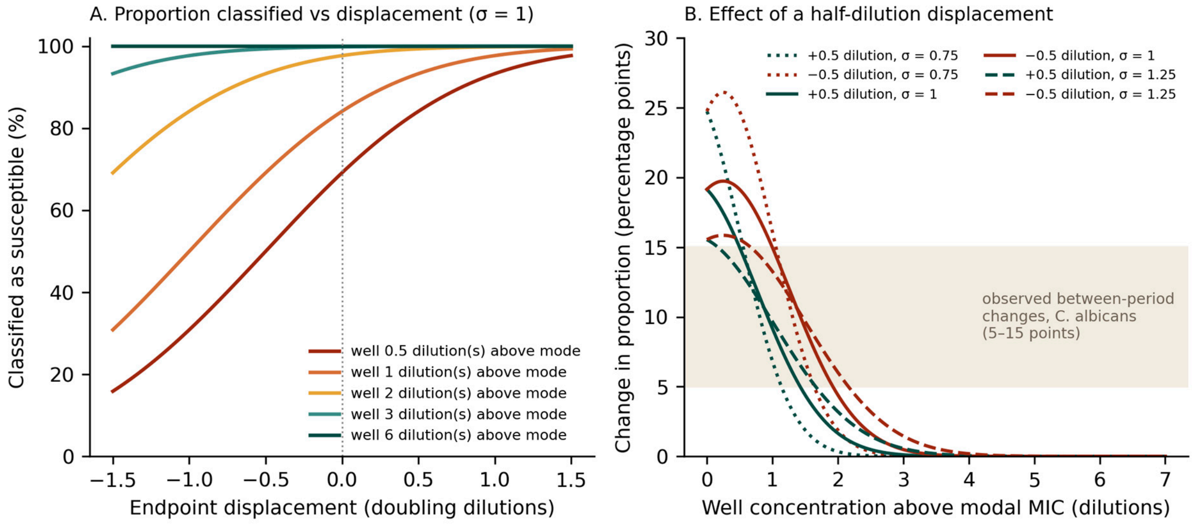

Figure S1. Sensitivity of a single-concentration categorical endpoint to displacement. (A) Proportion of isolates classified as susceptible as a function of the displacement of the effective endpoint, for wells placed 0.5, 1, 2, 3 and 6 doubling dilutions above the modal MIC (σ = 1). (B) Absolute change in the proportion classified after a displacement of ±0.5 dilution, as a function of the distance of the well above the modal MIC, for three widths of the MIC distribution. The shaded band marks the range of between-period changes observed for the four topical agents in *C. albicans* in the present study.

Supplementary Table S5

Table S5. Kit lots and control-strain acceptance testing for 2024 and 2025. Every lot was tested with *Candida parapsilosis* ATCC 22019 before being placed in use.

Year of use	Kit lot (Integral System YEASTS Plus, ref. 71822)	Kit lot expiry	Control strain lot (C. parapsilosis ATCC 22019)	Control lot expiry	Acceptance result
2024	020124502	22 May 2025	715837	12 Feb 2025	No deviation
	050324504	21 Aug 2025	715837	12 Feb 2025	No deviation
	053124505	20 Sep 2025	715837	12 Feb 2025	No deviation
2025	090524505	25 Dec 2025	155849	4 Feb 2026	No deviation
	010725505	9 Apr 2026	155849	4 Feb 2026	No deviation
	021425507	6 Jun 2026	174153	25 Mar 2026	No deviation

Year of use	Kit lot (Integral System YEASTS Plus, ref. 71822)	Kit lot expiry	Control strain lot (C. parapsilosis ATCC 22019)	Control lot expiry	Acceptance result
	032125513	9 Jul 2026	174153	25 Mar 2026	No deviation
	041725506	6 Aug 2026	235094	17 Aug 2026	No deviation
	072525511	14 Nov 2026	235094	17 Aug 2026	No deviation
	092925507	14 Jan 2027	235094	17 Aug 2026	No deviation
	103125513	18 Feb 2027	235094	17 Aug 2026	No deviation

Lot numbers and expiry dates are transcribed from the laboratory's kit acceptance records. Each kit lot comprises the test panel, the saline diluent and the xylose disk, which carry their own component lot numbers (for example, kit lot 032125513 contains panel lot 031125002, saline lot 040925001 and xylose disk lot 031125153). Acceptance testing consists of identification and categorical susceptibility reading of the reference strain after 48 h at 36 ± 1 °C; the laboratory's non-conformity register records no deviation from the expected result for any lot over the eight-year surveillance period. Records for 2024 and 2025 were retrieved for this analysis; those for earlier years are held in the institutional central archive. The manufacturer's certificate of analysis supplied with each lot documents identification and susceptibility to all ten antifungals of the panel with a reference *C. albicans* strain. Package insert revision 4 (25 July 2012) was supplied with the lots listed. Reading logs identifying the operator of individual clinical results were not retained.
